# Two machine learning methods identify a metastasis-related prognostic model that predicts overall survival in medulloblastoma patients

**DOI:** 10.18632/aging.103923

**Published:** 2020-11-05

**Authors:** Kui Chen, Bingsong Huang, Shan Yan, Siyi Xu, Keqin Li, Kuiming Zhang, Qi Wang, Zhongwei Zhuang, Liang Wei, Yanfei Zhang, Min Liu, Hao Lian, Chunlong Zhong

**Affiliations:** 1 Department of Neurosurgery, Shanghai East Hospital, Tongji University School of Medicine, Shanghai 200120, P.R. China; 2 Huamu Community Health Service Center, Shanghai 201204, P.R. China

**Keywords:** medulloblastoma, machine learning, prognostic model, overall survival

## Abstract

Approximately 30% of medulloblastoma (MB) patients exhibit metastasis at initial diagnosis, which often leads to a poor prognosis. Here, by using univariate Cox regression analysis, two machine learning methods (Lasso-penalized Cox regression and random survival forest-variable hunting (RSF-VH)), and multivariate Cox regression analysis, we established two metastasis-related prognostic models, including the 47-mRNA-based model based on the Lasso method and the 21-mRNA-based model based on the RSF-VH method. In terms of the results of the receiver operating characteristic (ROC) curve analyses, we selected the 47-mRNA metastasis-associated model with the higher area under the curve (AUC). The 47-mRNA-based prognostic model could classify MB patients into two subgroups with different prognoses. The ROC analyses also suggested that the 47-mRNA metastasis-associated model may have a better predictive ability than MB subgroup. Multivariable Cox regression analysis demonstrated that the 47-mRNA-based model was independent of other clinical characteristics. In addition, a nomogram comprising the 47-mRNA-based model was built. The results of ROC analyses suggested that the nomogram had good discrimination ability. Our 47-mRNA metastasis-related prognostic model and nomogram might be an efficient and valuable tool for overall survival (OS) prediction and provide information for individualized treatment decisions in patients with MB.

## INTRODUCTION

Medulloblastoma (MB) is the most frequent type of malignant pediatric brain tumor and comprises at least four distinct molecular subgroups: WNT, SHH, Group 3, and Group 4 [1, 2]. The presence of metastatic disease often results in a less favorable outcome for MB patients, and unfortunately, approximately 25-33% of MB patients present with metastases at the time of diagnosis [3, 4]. Currently, the standard protocol, including surgery followed by craniospinal radiation and chemotherapy, achieves an overall survival (OS) rate of about 85% at 5 years for standard-risk patients with MB [5–7]. However, a number of survivors suffer from serious treatment-related effects of radiotherapy and cytotoxic chemotherapy, resulting in a decline in cognition and intellect, endocrine disorders and an increased incidence of secondary cancers [8, 9]. In addition, some MB patients receive unnecessary or excessive therapies, while others may be faced with metastasis or recurrence because of a lack of appropriate treatment. The risk stratification of MB patients is mainly based on age at diagnosis, size of the residual disease, metastatic status, histology, subgroup, and some cytogenetic biomarkers [4, 10, 11]. The current therapies and the risk stratification used since the late 1980s pose tremendous challenges [10]. The survival rate of MB patients has been stagnant for approximately 30 years despite the multipronged approach to therapy [12]. These limitations have prompted a search for more precise and comprehensive biomarkers for the discrimination of MB patients to improve precision MB treatment.

With the advances of high-throughput microarray and RNA sequencing technologies, an increasing number of metastasis-related prognostic signatures have been identified in various types of cancers [13–18]. A six-gene metastasis signature has been reported to be robust for predicting the survival of hepatocellular carcinoma patients in multicenter cohorts [15]. Another study showed that some novel tissue- and serum-based metastasis-specific microRNA biomarkers could be clinically applicable to predict prognosis in colorectal cancer [13]. In addition, a lymph-node-metastasis-related gene signature had stronger predictive power than other clinical information for the prognostic evaluation of esophageal cancer [17]. These studies suggest that metastasis-related signatures might serve as potentially accurate biomarkers for predicting the outcome of cancer patients. Therefore, searching for a metastasis-related biomarker signature may have concrete prognostic and predictive value in the management of MB. Moreover, the identification of metastasis-related molecular markers might pave the way for precisely targeted metastasis-related molecular therapies for MB.

In the present study, we focused on the mRNA expression profiles of large cohorts of patients with MB from the Gene Expression Omnibus (GEO) database. The differentially expressed genes (DEGs) were screened by analyzing the gene expression data between MB tissues with and without metastasis. Then, by employing Cox regression analysis and two machine learning algorithms, including the Lasso-penalized Cox regression model and random survival forest-variable hunting (RSF-VH) algorithm, we identified a metastasis-related prognostic signature that can accurately predict survival in MB patients. Moreover, the metastasis signature was a survival-related factor independent of well-known clinical characteristics. Finally, we built a predictive nomogram that showed good discrimination ability and was clinically useful. Overall, the metastasis signature and nomogram may be reliable and practical prognostic tools for OS evaluation and might facilitate individualized therapy for MB patients with different risks of disease.

## RESULTS

### Development and validation of a 47-mRNA metastasis-related prognostic model

**Figure 1:**
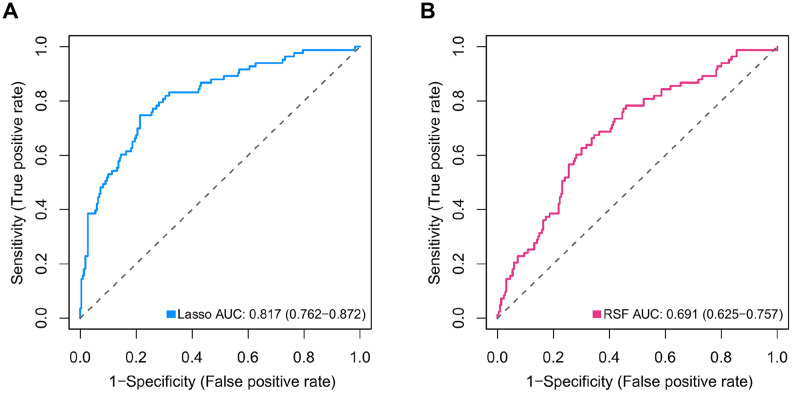
**Comparison of the predictive power of the 47-mRNA metastasis-related model based on Lasso-penalized Cox regression and the 21-mRNA metastasis-related model based on random survival forest-variable hunting (RSF-VH).** The receiver operating characteristic (ROC) curves of the 47-mRNA-based model based on Lasso-penalized Cox regression (**A**) and the 21-mRNA-based model based on RSF-VH (**B**).

Differential expression analysis using metastatic status as the group variable identified a total of 2,429 DEGs (adjusted *P* < 0.2). To define the association of the DEGs with the OS of MB patients, univariate Cox regression analysis was conducted, and the results revealed that 307 of the 2,429 DEGs were significantly related to OS in MB patients. Then, we employed the Lasso-penalized Cox regression and RSF-VH methods to identify the DEGs with the greatest prognostic value in which we required the selected prognostic DEGs to appear > 100 times out of 1,000 repetitions. Finally, by using multivariable Cox regression analysis, a 47-mRNA metastasis-related prognostic model based on Lasso-penalized Cox regression and a 21-mRNA metastasis-related prognostic model based on RSF-VH were established. Supplementary Tables 1 and 2 show the multivariate Cox regression coefficients of the genes in the 47-mRNA metastasis-related prognostic model and the 21-mRNA metastasis-related prognostic model, respectively. In addition, Supplementary Tables 3 and 4 show the repeat occurrence frequencies of the genes in the 47-mRNA metastasis-related prognostic model and the 21-mRNA metastasis-related prognostic model, respectively. To investigate the predictive efficiency of the afore-mentioned two metastasis-related prognostic models, the receiver operating characteristic (ROC) curve analysis was performed. The resulting area under the curve (AUC) of the 47-mRNA metastasis-associated prognostic model was 0.817 (95% CI: 0.762-0.872) (Figure 1A), while the resulting AUC of the 21-mRNA metastasis-associated prognostic model was 0.691 (95% CI: 0.625-0.757) (Figure 1B). Therefore, the 47-mRNA metastasis-associated prognostic model with the higher AUC was selected for further analysis.

**Figure 2:**
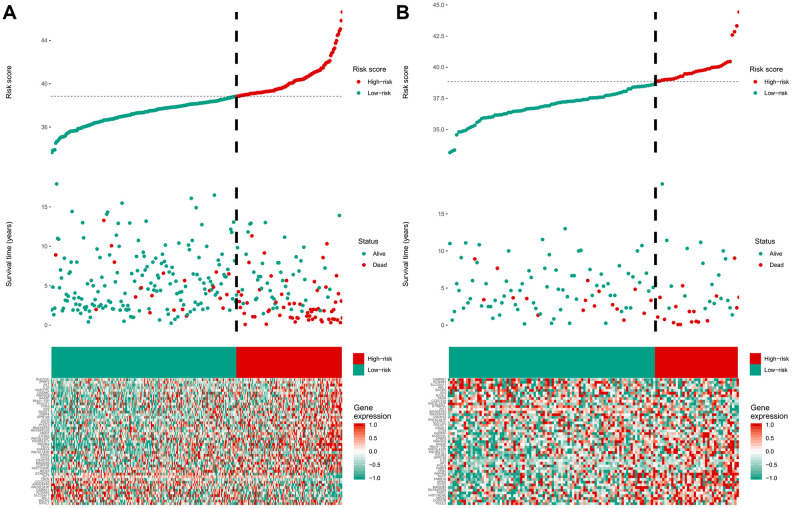
****The distribution of the risk score, overall survival (OS), OS status, and heatmap of the 47-mRNA metastasis-related model in the discovery set (**A**) and validation set (**B**).

**Figure 3:**
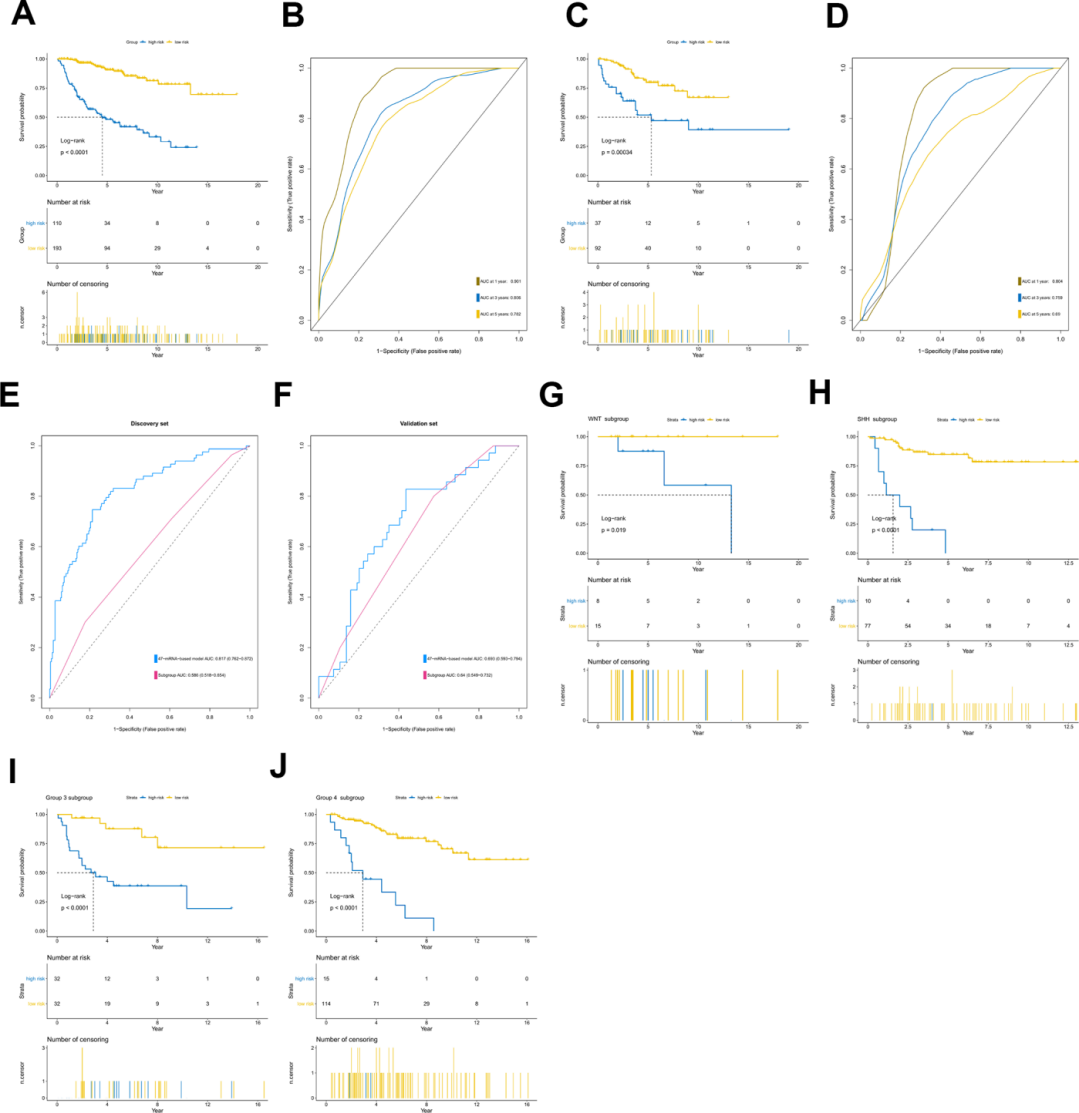
**Prognostic value of the 47-mRNA metastasis-related model.** The Kaplan-Meier (K-M) curves show the OS of the high- and low-risk patients with MB classified by the optimal cutoff value. (**A**) K-M curves for the discovery set. (**B**) ROC curves for the 47-mRNA-based model in the discovery set. (**C**) K-M curves for the validation set. (**D**) ROC curves for the 47-mRNA-based model in the validation set. (**E**) The comparison of the area under the ROC of the 47-mRNA-based model versus that of subgroup in the discovery set. (**F**) The comparison of the area under the ROC of the 47-mRNA-based model versus that of subgroup in the validation set. (**G**) K-M curves showing the OS for the high- and low-risk patients with WNT MB using the 47-mRNA-based model in the discovery set. (**H**) K-M curves showing the OS for the high- and low-risk patients with SHH MB using the 47-mRNA-based model in the discovery set. (**I**) K-M curves showing the OS for the high- and low-risk patients with group 3 MB using the 47-mRNA-based model in the discovery set. (**J**) K-M curves showing the OS for the high- and low-risk patients with group 4 MB using the 47-mRNA-based model in the discovery set.

**Figure 4:**
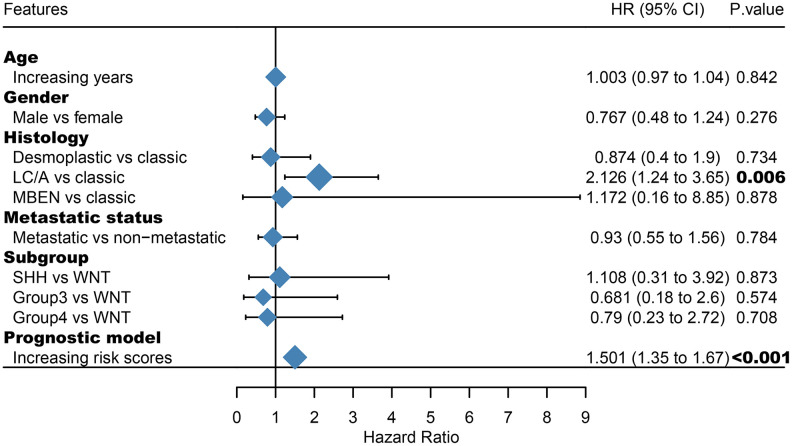
**Multivariate Cox regression analysis incorporating the 47-mRNA metastasis-related model and known prognostic clinical characteristics.** LC/A, large cell/anaplastic; MBEN, medulloblastoma with extensive nodularity.

Among the genes in the 47-mRNA metastasis-related prognostic model, 31 genes (AK7, ARL1, ARSG, BACH2, C9orf153, COPS7B, CPB2, EIF2B3, FABP4, GAGE1, GPR126, GUCY2C, GYG2, HIST1H2AE, ICOS, IDI2, MAGEB5, MEIS2, NTHL1, NUP210L, POLN, POU1F1, PSORS1C1, RN7SKP226, RN7SL432P, RNA5SP53, SAA3P, STXBP5L, TBCC, ZIC1, and ZPBP2) had positive coefficients, indicating an association between their higher expression levels and shorter OS, while the higher expression levels of the remaining 16 genes with negative coefficients (ARHGEF40, CAMKK1, CCDC125, FAM81A, GSDMC, IL22, KCNAB3, MDN1, PAPPA2, POLE3, RN7SL187P, RN7SL581P, RNASE9, RNU1-75P, SLC25A11, and TBCK) may correlate with longer OS. The distributions of the 47-gene-based risk scores, OS, survival status, and 47-gene expression profiles of the MB patients in the discovery set and validation set are shown in Figure 2A, 2B. The 31 risk-related mRNAs tended to be more highly expressed in the high-risk group, whereas the 16 protective mRNAs tended to exhibit higher expression in the low-risk group. K-M survival analysis showed that in the discovery set, patients in the high-risk group (n = 110) had a significantly shorter OS than those in the low-risk group (n = 193; *P* < 0.0001; Figure 3A). Similar results were observed in the validation set (*P* = 0.00034; Figure 3C). To evaluate the predictive performance of the 47-mRNA metastasis-related prognostic model, we performed time-dependent ROC curve analysis. The AUCs of the metastasis-related prognostic model were 0.901 at 1 year, 0.806 at 3 years, and 0.782 at 5 years for the discovery set (Figure 3B), and 0.804 at 1 year, 0.759 at 3 years, and 0.69 at 5 years for the validation set (Figure 3D). All AUCs exceeded 0.6, indicating that the metastasis-related forecast model had a good performance for OS prediction in MB patients. The 47-mRNA metastasis-related prognostic model had a better predictive performance than MB subgroup in the discovery set (0.817 vs 0.586) (Figure 3E) and in the validation set (0.693 vs 0.64) (Figure 3F). When we employed the 47-mRNA metastasis-related prognostic model to predict the survival of patients in each MB subgroup, we found that there were statistically significant differences in OS between the high-risk group and the low-risk group (Figure 3G–3J). In addition, the results of multivariable Cox regression analysis revealed that the metastasis-associated prognostic model was a powerful and independent prognostic factor related to OS (Figure 4).

### Weighted gene co-expression network analysis and gene ontology enrichment analysis for identifying the pathways significantly associated with the 47-mRNA-based risk score model

**Figure 5:**
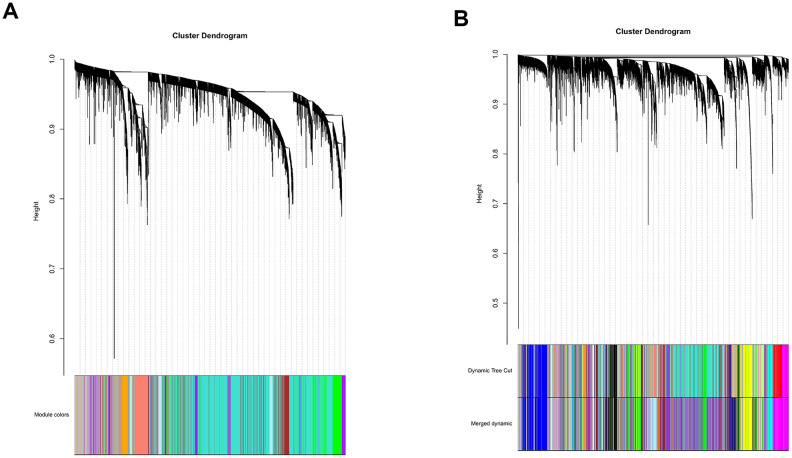
**Establishment of co-expression modules of MB.** The colored bars below the clustering dendrogram represent the original modules (**A**) and merged modules (**B**). Fifty-five modules were generated by the Dynamic Tree Cut method. Forty-one modules were identified after merging according to the module dissection threshold.

**Figure 6:**
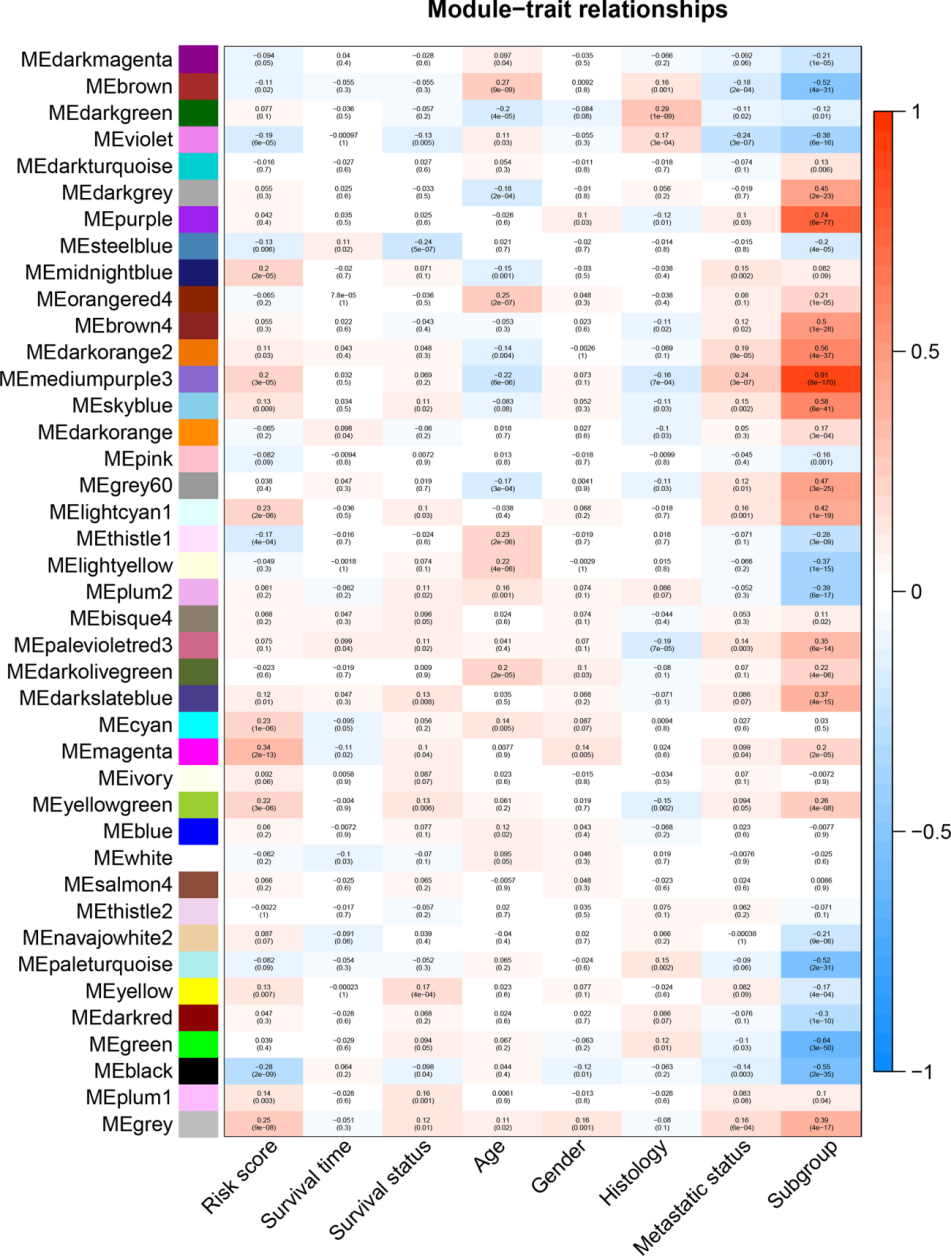
**Heatmap of the correlation between gene modules and clinical traits of MB.** Each row in the heatmap corresponds to a module, and each column in the heatmap corresponds to a specific clinical characteristic. Each cell contains the corresponding correlation coefficient and *p*-value.

**Figure 7:**
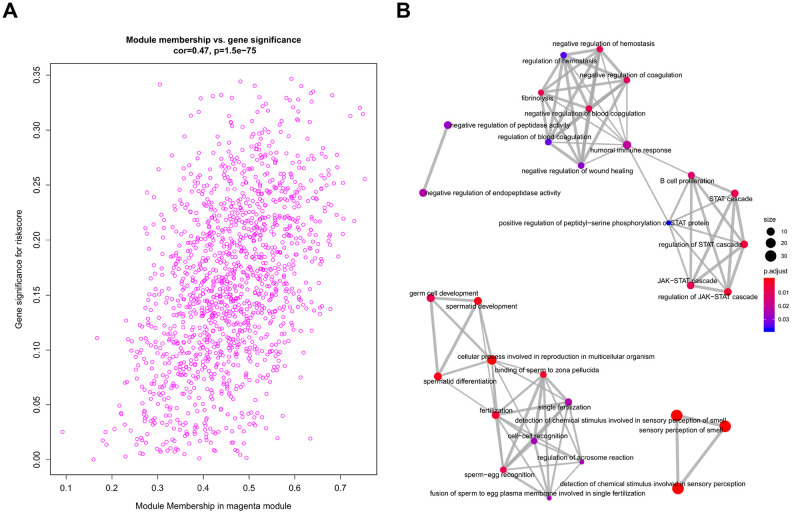
**Functional annotation for magenta module.** (**A**) Scatter plot of module eigengenes associated with risk score in the magenta module. (**B**) GO analysis involved in the co-expression magenta module.

All genes from entire MB dataset were applied to construct a gene co-expression network using weighted gene co-expression network analysis (WGCNA). The original 55 modules were obtained with Dynamic Tree Cut method (Figure 5A). The module dissection threshold was set at 0.3 to merge correlated modules and 41 modules were finally generated (Figure 5B). The correlations between co-expression modules and clinical phenotypes were calculated and visualized through a heatmap (Figure 6). The scatterplot of gene significance (GS) for the 47-mRNA-based risk score model vs. module membership (MM) was plotted in the co-expression magenta module (Figure 7A). Gene ontology (GO) enrichment analysis of hub genes revealed a significantly relationship between sensory perception of smell, cellular process involved in reproduction, and regulation of STAT cascade and the 47-mRNA-based risk score model (Figure 7B).

### Construction and validation of a metastasis-related nomogram

**Figure 8:**
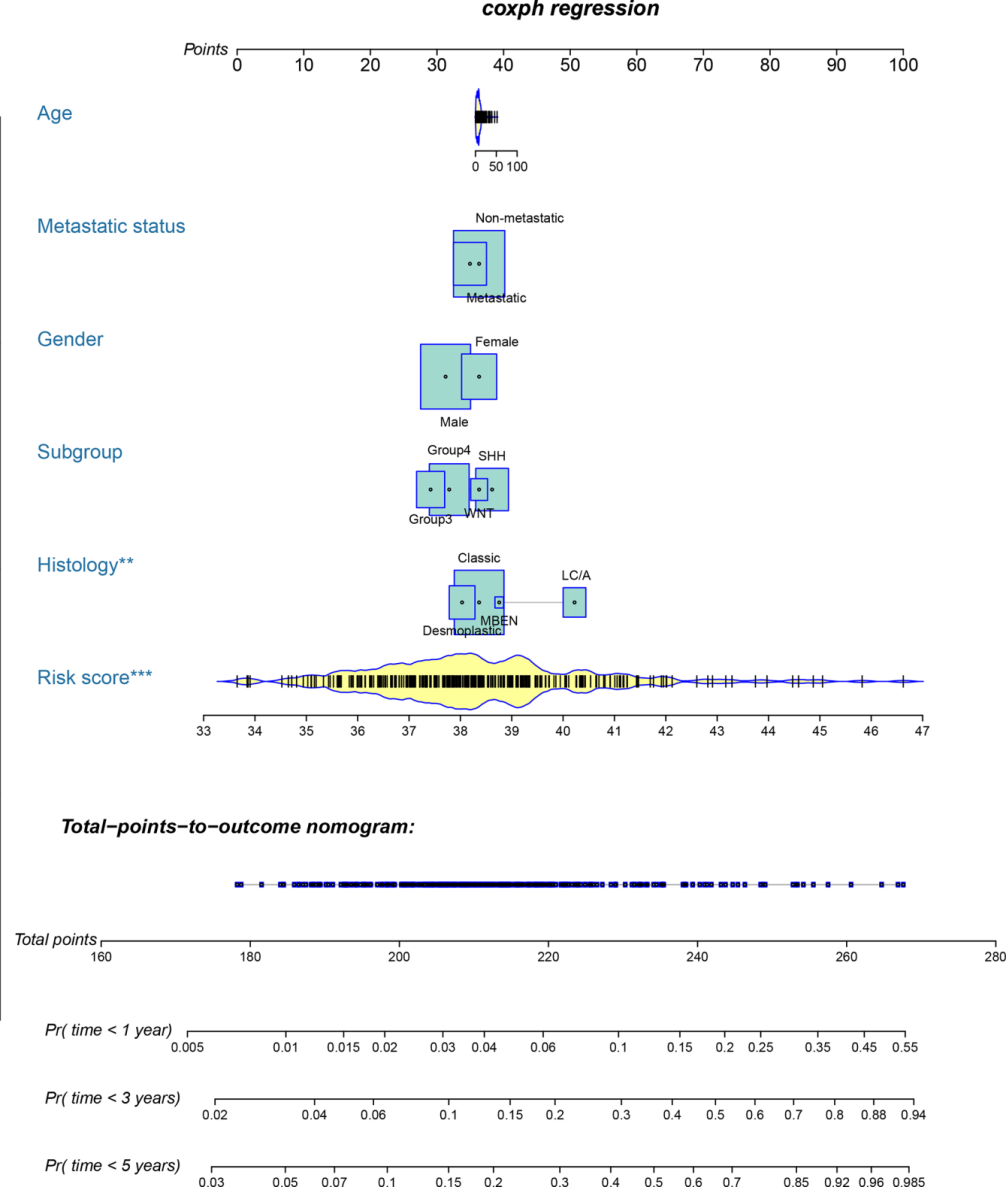
**Nomogram for predicting 1-, 3-, and 5-year OS in patients with MB.**

**Figure 9:**
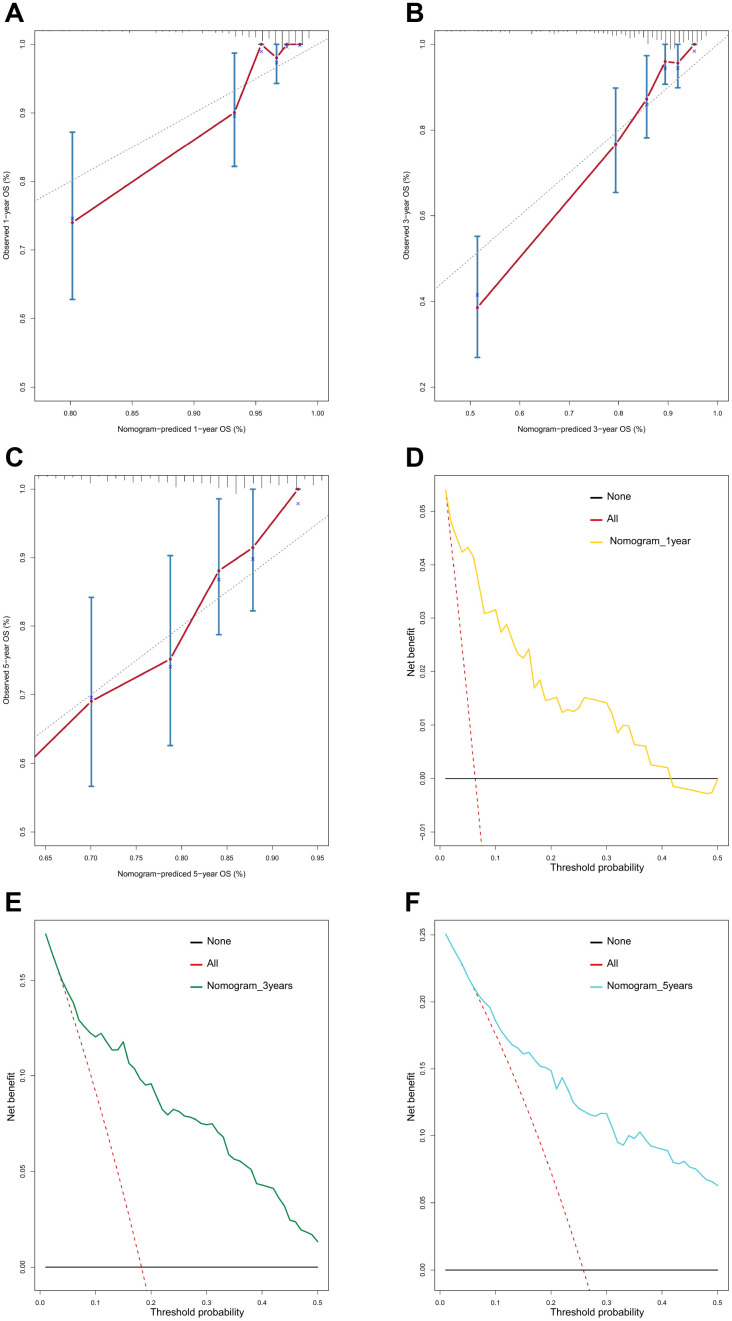
**Calibration curves and decision curve analysis (DCA) of the nomogram.** Calibration curves of the nomogram for predicting OS at 1 year (**A**), 3 years (**B**), and 5 years (**C**). DCA of the nomogram for predicting OS at 1 year (**D**), 3 years (**E**), and 5 years (**F**).

**Figure 10:**
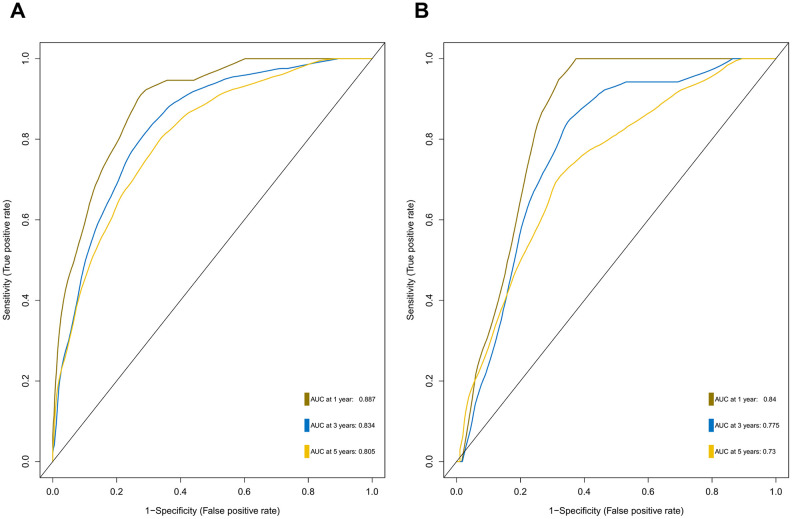
****ROC curves for the nomogram in the discovery set (**A**) and validation set (**B**).

To provide clinicians with a quantitative method that could predict the probability of 1-, 3-, and 5-year OS in patients with MB, a metastasis-associated nomogram was generated by integrating the 47-mRNA metastasis-related prognostic model and five clinicopathological factors (Figure 8). Calibration plots showed that the metastasis-related nomogram performed well compared with the ideal curve (the 45-degree line) (Figure 9A–9C). Decision curve analysis (DCA) indicated that if the threshold probability of patients or doctors is more than 10%, then utilizing the metastasis-related nomogram to predict the probability of 1-, 3-, and 5-year OS adds more net benefit than the treat-none scheme or the treat-all-patients scheme (Figure 9D–9F). The AUCs of the metastasis-related nomogram were 0.887 at 1 year, 0.834 at 3 years, and 0.805 at 5 years for the discovery set (Figure 10A), and 0.84 at 1 year, 0.775 at 3 years, and 0.73 at 5 years for the validation set (Figure 10B).

## DISCUSSION

Although MB consists of four primary molecular subgroups with disparate clinical outcomes, molecular markers that could precisely predict survival in MB patients are still lacking. Given that nonmetastatic and metastatic patients with MB often have distinct outcomes, metastasis-associated mRNAs may be accurate predictors of outcome in MB patients. In our study, the differentially expressed genes between metastatic and nonmetastatic MB tissues were screened, and univariate Cox analysis, two different machine learning algorithms including Lasso-penalized Cox regression and RSF-VH, and multivariate Cox analysis were performed to construct two metastasis-related prognostic models (the 47- mRNA metastasis-related model based on Lasso Cox regression and the 21-mRNA metastasis-related model based on RSF-VH). According to the predictive performance, we chose the 47-mRNA metastasis-associated model based on Lasso Cox regression with the higher AUC. More recently, machine learning approaches have been successfully utilized for identifying novel diagnostic molecular markers, tracking cancer development, predicting cancer prognosis and monitoring treatment responses to allow the accurate classification of cancer [19]. Lasso and RSF are two common machine learning methods used for building cancer-related prognostic models. Several recent studies showed that in the case of low data dimensions, linear models such as Lasso regression can separate samples more ideally, whereas more complex machine learning models such as random forest are more prone to overfitting, leading to a less precise prediction [20–22]. Thus, considering that the sample size used in the present study is relatively small, Lasso Cox regression may be an appropriate method to establish MB-related prognostic models.

The 47-mRNA metastasis-related model developed in this study categorized MB patients into low- and high-risk groups with significantly different OS outcomes in the discovery and validation cohorts. The clinicians could design the MB patients’ treatment plans based on the predicted outcome of the metastasis-associated model to achieve individualized treatment of patients with MB. Strategies should be developed to prevent metastasis or detect MB metastases as early as possible in high-risk MB populations.

Among the 47 genes of the metastasis-related model based on Lasso Cox regression, except for ZIC1 [23, 24], the other 46 genes were either poorly investigated or have not been reported in MB. In addition, five genes involved in the 47-mRNA metastasis-related model, including FABP4 [25, 26], GUCY2C [27–29], MEIS2 [30, 31], POU1F1 [32–34], and SLC25A11 [35] have been reported to be related to the metastasis of other human cancers. Although the roles of these five genes in MB are presently unclear, our results suggest that they deserve further biological and mechanistic investigation. Interestingly, we found that seven pseudogenes, including RN7SKP226, RN7SL187P, RN7SL432P, RN7SL581P, RNA5SP53, RNU1-75P, and SAA3P, were included in the 47-mRNA metastasis- related model, indicating that expression analysis of these pseudogenes might become a new paradigm for investigating MB mechanisms and discovering prognostic biomarkers in MB. Therefore, the 47-mRNA metastasis-related model may offer potential therapeutic targets for MB treatment.

Furthermore, we demonstrated that the 47-mRNA metastasis-related model remained an independent prognostic factor after adjusting for other clinical characteristics. This finding suggests that a comprehensive model incorporating the 47-mRNA metastasis-related model and other clinicopathological factors may achieve a more reliable and favorable OS prediction efficacy for MB patients. Therefore, we built a nomogram that combined the 47-mRNA prognostic model and other clinical features (age, sex, histology, metastatic status, and subgroup). The calibration curves showed that the actual OS corresponded closely with the predicted OS, indicating that the predictive performance of the metastasis-related nomogram was good. DCA demonstrated that the metastasis-related nomogram was clinically useful. According to the results of ROC analyses, this nomogram showed good discrimination ability. Thus, our nomogram could be a promising tool for facilitating patient counselling, treatment decision-making, and follow-up scheduling.

However, our study had some limitations. First, our 47-mRNA metastasis-related model and nomogram need further validation in multicenter, large-scale, prospective studies. Second, functional and mechanistic studies on the 47 genes alone and in combination should be performed to support their clinical application. Third, information on radiotherapy protocols, chemotherapeutic regimens, patient neurological/clinical status, and cytogenetic aberrations is not available in the MB cohort included in the present study. Finally, we constructed the 47-mRNA metastasis-related model based on the gene expression data without considering the DNA methylation, mutation, or other genetic events of genes that likely have an effect on the metastasis of MB.

In summary, our findings indicate that the 47-mRNA metastasis-related prognostic model derived from Lasso-penalized Cox regression might be a reliable and useful tool for predicting OS in MB patients. A nomogram comprising the metastasis-related prognostic model may assist clinicians in selecting personalized therapeutic regimens for MB patients.

## MATERIALS AND METHODS

### Data source

MB gene expression data were directly downloaded from the GEO GSE85218 dataset (http://www.ncbi.nlm.nih.gov/geo/query/acc.cgi?acc=GSE85218) [36]. The corresponding clinical information was obtained from the supplementary data in the relative literature [36]. Then, MB patients with no information on at least one of the following clinical characteristics were excluded from further analysis: age, sex, metastatic status, histology, survival time, and survival state. Finally, a total of 432 MB patients were included in this study; the median age was 8.00 years (range, 0.24 to 52.00 years) and the median OS was 4.06 years (range, 0.08 to 19.03 years). These 432 MB patients were randomly divided into a discovery set (70%) and validation set (30%) by utilizing the createDataPartition function of the caret package for R software. The distribution of the baseline clinical characteristics of the two groups was balanced (all *P* values > 0.05). The clinical information of these MB patients is summarized in Supplementary Table 5.

### Construction and validation of a metastasis-associated gene signature

In the discovery set, DEGs between MB tissues from patients with and without metastatic disease were calculated using the limma package for R software. The DEGs with an adjusted *P* value of < 0.2 were considered for downstream analysis. Next, univariate Cox proportional hazards regression analyses were performed to investigate the association between the OS of MB patients in the discovery set and the expression level of each DEG. In the univariate Cox regression analyses, the genes whose parameter *P* values were < 0.05 were selected for subsequent analysis. To further select primary predictive features, we applied two well-established machine learning algorithms (Lasso-penalized Cox regression and RSF-VH) on the discovery set. Within these two analyses, we subsampled the discovery set at a ratio of 7:3 with 1,000 replacements and selected the prognostic DEGs with repeat occurrence frequencies of more than 100. Then, two metastasis-related risk score staging models (one derived from Lasso-penalized Cox regression and another derived from RSF-VH) were constructed based on a linear combination of the regression coefficient obtained from the multivariate Cox regression analysis (β_i_) multiplied by its expression level (expr_i_). The formula for computing the risk scores of these two prognostic models is described as follows:

$$\text{Risk}\;\text{score}=\sum_{\text{i}=1}^{\text{n}}\left(\beta_{\text{i}}*{\text{expr}}_{\text{i}}\right)$$

The area under the curve (AUC) of the ROC curve was calculated to assess the prediction efficiency of the two metastasis-related prognostic models by using the pROC package for R software. The metastasis-associated risk score model with the higher AUC was kept for subsequent analysis. MB patients in the discovery set were classified into high and low risk score groups according to the optimal risk score cutoff point yielded by utilizing the surv_cutpoint function of the survminer package for R software. We also performed ROC analyses to compare the specificity and sensitivity of OS prediction based on the metastasis-related prognostic model and MB subgroup. Given that the validation set size is small (WNT subgroup, n=12; SHH subgroup, n=35; Group 3, n=17; Group 4, n=65), we only employed the metastasis-related prognostic model to predict survival of patients for each MB subgroup in the discovery set. To test whether the metastasis-related prognostic model was independent of other clinical features (including age, sex, histology, metastatic status, and molecular subgroup), multivariate Cox regression analyses were performed. In the validation set, we used the same risk score formula and cutoff value and divided the MB patients into high- and low-risk groups to test the robustness of the metastasis-related risk score model. The survival difference between the low- and high-risk groups in each set was evaluated by the Kaplan-Meier (K-M) method and compared with the log-rank test.

### WGCNA and GO enrichment analysis for discovering the pathways significantly correlated with the 47-mRNA-based risk score model

The expression data of all genes and clinical data (risk score, survival time, survival status, age, gender, histology, metastatic status, and subgroup) in entire MB cohort were included in the WGCNA and analyzed by using the WGCNA package for R software [37]. The all genes were classified into some co-expression modules utilizing an appropriate soft-thresholding parameter β which was calculated by using the pickSoftThreshold function. The Eigengenes for each co-expression module were calculated and correlated modules were merged according to the module dissection threshold. By calculating the correlation between co-expression modules and clinical features by the module-trait relationship of WGCNA, we could screen the module most associated with the clinical trait we were interested in. In this study, the 47-mRNA-based risk score model was selected as the interested clinical trait for subsequent analysis. After the interesting module was chosen, we defined the cor.geneTraitSignificance > 0.2 (the correlation between the gene expression profile and the module eigengene) and the cor.geneModule Membership > 0.4 (the correlation between a certain clinical phenotype and the gene) as the threshold for screening hub genes in a module. GO enrichment analysis was performed by using the clusterProfiler package for R software.

### Establishment and validation of a predictive nomogram

A metastasis-related nomogram was constructed to predict 1-, 3-, and 5-year OS for MB patients by combining the metastasis-related risk score model with clinical variables (age, sex, histology, metastatic status, and subgroup) by using the regplot package for R software. Subsequently, validation, comprising the discrimination ability and predictive accuracy of the nomogram, was performed. Time-dependent ROC curve analyses, which were conducted with the R package “survivalROC”, were performed to assess the discrimination ability of the nomogram. The predictive accuracy of the nomogram was determined using the calibration plots, which were generated with the R package “rms”. Additionally, decision curve analysis was conducted to assess the clinical usefulness of the nomogram by quantifying the net benefits for a range of threshold probabilities using the “stdca.R” package [38].

### Statistical analysis

All statistical analyses were executed by R 3.5.2. Lasso Cox regression analysis and RSF-VH were performed with the R package “glmnet” and “randomForestSRC”, respectively. For survival analyses including the K-M method and Cox regression, a two-sided *P* value < 0.05 was considered statistically significant. The adjusted *P* values for multiple testing were calculated by using the Benjamini-Hochberg method.

### Data accessibility

The data that support the findings of the current study are available from the corresponding authors on reasonable request.

## Supplementary Material

Supplementary Tables
