# Supplementary material for: Two machine learning methods identify a metastasis-related prognostic model that predicts overall survival in medulloblastoma patients
Source: Aging (Albany NY). 2020 Nov 5;12(21):21481–503. doi: 10.18632/aging.103923 (PMC7695392; doi:10.18632/aging.103923)
Supplement: Supplementary Tables [file aging-12-103923-s001..pdf]

SUPPLEMENTARY TABLES

Supplementary Table 1. The multivariate Cox regression coefficients of the genes in the 47-mRNA metastasis-related prognostic model.

Gene name	Coefficient
AK7	0.179608
ARHGEF40	-0.62077
ARL1	0.670076
ARSG	0.31238
BACH2	0.387613
C9orf153	0.759155
CAMKK1	-0.42176
CCDC125	-0.50066
COPS7B	0.417233
CPB2	0.490751
EIF2B3	0.244405
FABP4	0.084767
FAM81A	-0.09479
GAGE1	0.085492
GPR126	0.176501
GSDMC	-0.03405
GUCY2C	0.926616
GYG2	0.545351
HIST1H2AE	0.757313
ICOS	0.48971
IDI2	0.382761
IL22	-0.27895
KCNAB3	-0.17314
MAGEB5	0.560332
MDN1	-0.4732
MEIS2	0.233501
NTHL1	1.20003
NUP210L	0.776257
PAPPA2	-0.05873
POLE3	-0.64073
POLN	0.177848
POU1F1	0.06912
PSORS1C1	1.19728
RN7SKP226	0.206219

RN7SL187P	-0.62164
RN7SL432P	0.854117
RN7SL581P	-1.14738
RNA5SP53	0.854826
RNASE9	-0.09789
RNU1-75P	-0.51289
SAA3P	0.462747
SLC25A11	-0.39191
STXBP5L	0.008379
TBCC	0.709829
TBCK	-0.46125
ZIC1	0.077023
ZPBP2	1.18368

Supplementary Table 2. The multivariate Cox regression coefficients of the genes in the 21-mRNA metastasis-related prognostic model.

Gene name	Coefficient
AK7	0.003188
ARHGEF40	-0.81575
FABP4	0.220511
FAM81A	0.262222
GPR126	0.0707
ICT1	-0.0832
IDI2	0.887522
LRRC45	0.913915
LUZP4	0.160284
MEIS2	0.274243
OR2W1	0.888631
PPIL1	-0.62113
PSMG3	0.348928
RN7SKP226	0.395319
RNA5SP318	-0.16243
RNU4-13P	0.072429
RNU5A-2P	0.495492
SCN8A	-0.06987
SLC8B1	0.088436
STXBP5L	-0.20713
TBCC	0.456662

Supplementary Table 3. The repeat occurrence frequencies of the genes in the 47-mRNA metastasis-related prognostic model based on Lasso-penalized Cox regression.

Gene name	Repeat occurrence frequency
NTHL1	616
SLC25A11	596
IDI2	586
ARHGEF40	509
MEIS2	440
ZPBP2	432
CCDC125	407
RN7SKP226	391
RNA5SP53	387
KCNAB3	372
HIST1H2AE	370
RN7SL581P	366
GPR126	355
FABP4	337
FAM81A	334
PSORS1C1	333
BACH2	303
GYG2	289
NUP210L	283
RN7SL187P	251
RNU1-75P	248
POLE3	247
ZIC1	235
AK7	211
SAA3P	208
ARSG	206
CAMKK1	205
EIF2B3	201
POLN	183
TBCC	179
MAGEB5	171
PAPPA2	165
CPB2	155
COPS7B	154
C9orf153	153
STXBP5L	152
GAGE1	142
MDN1	137

POU1F1	137
ARL1	121
ICOS	118
RN7SL432P	115
RNASE9	114
IL22	111
TBCK	109
GSDMC	108
GUCY2C	108

Supplementary Table 4. The repeat occurrence frequencies of the genes in the 21-mRNA metastasis-related prognostic model based on RSF-VH.

Gene name	Repeat occurrence frequency
ICT1	229
ARHGEF40	201
FABP4	164
RNA5SP318	151
IDI2	146
FAM81A	141
STXBP5L	133
LUZP4	127
SCN8A	120
TBCC	120
OR2W1	116
PSMG3	115
LRRC45	113
RNU5A-2P	109
AK7	108
RNU4-13P	107
SLC8B1	106
RN7SKP226	105
GPR126	104
PPIL1	104
MEIS2	102

Supplementary Table 5. Comparison of distribution of clinical characteristics between the discovery and validation set.

Parameter	Discovery set (n= 303)	Validation set (n=129)	*p*-value
**Age** (mean (SD))	8.78 (7.74)	10.30 (8.21)	0.066
**Gender** (n (%))			
Female	100 (33.0)	54 (41.9)	0.099
Male	203 (67.0)	75 (58.1)	
**Histology** (n (%))			
Classic	201 (66.3)	91 (70.5)	0.052
Desmoplastic	54 (17.8)	15 (11.6)	
LC/A	42 (13.9)	15 (11.6)	
MBEN	6 (2.0)	8 (6.2)	
**Metastatic status** (n (%))			
Non-metastatic	214 (70.6)	89 (69.0)	0.822
Metastatic	89 (29.4)	40 (31.0)	
**Subgroup** (n (%))			
Group 3	64 (21.1)	17 (13.2)	0.197
Group 4	129 (42.6)	65 (50.4)	
SHH	87 (28.7)	35 (27.1)	
WNT	23 (7.6)	12 (9.3)	

Abbreviations: SD, standard deviation; LC/A, large cell/anaplastic; MBEN, medulloblastoma with extensive nodularity.
